# Lessons learned: management of acute kidney injury during COVID-19

**DOI:** 10.1080/0886022X.2026.2637297

**Published:** 2026-03-05

**Authors:** Iyad Mansour, Swetha Rani Kanduri, Muna Danial Muneeb Omar, Ibrahim Sardar, Alicia Rachel Farr, Bijin Thajudeen

**Affiliations:** a Division of Nephrology, Banner University of Arizona, Tucson, AZ, USA; b Division of Nephrology/Department of Medicine, University of Texas Health, SanAntonio, TX, USA; c Division of Pulmonary and Critical care, Southern Arizona Veterans Health Care System, Tucson, AZ, USA; d College of Medicine, University of Arizona, Tucson, AZ, USA

The COVID-19 pandemic, caused by the novel coronavirus SARS-CoV-2, has had significant medical and financial impacts globally since its emergence in Wuhan, China. As of October 2025, the pandemic resulted in 784 million cases and 7.1 million fatalities [1]. While the primary focus of the virus is the respiratory system, it also poses risks to the kidneys, particularly in patients with preexisting renal conditions. The virus enters human cells through angiotensin converting enzyme 2 (ACE2) receptors, and in the kidneys, these ACE2 receptors are present in the tubular, glomerular, and endothelial cells, justifying the various functional and histological disorders associated with COVID-19 [2]. Kidney involvement in COVID-19 can lead to acute kidney injury (AKI) with an occurrence rate between 10% and 17% and the higher rates are observed among those with chronic kidney disease (CKD) or severe infections requiring intensive care [2]. The causes of AKI in these patients are multifactorial, often resulting from a combination of direct viral injury, a systemic inflammatory response, renal ischemia, acute interstitial nephritis, and hemodynamic changes due to respiratory distress or cardiac dysfunction [3]. In the context of COVID-19, several biomarkers are effective for detecting AKI, categorized into three groups: functional, damage, and stress biomarkers. Functional biomarkers like cystatin C assess kidney function. Damage biomarkers include kidney injury molecule-1, l-type fatty acid binding protein, interleukin-18, soluble urokinase-type plasminogen activator receptor, and neutrophil gelatinase-associated lipocalin. Stress biomarkers include tissue inhibitor of metalloproteinases-2 and insulin-like growth factor binding protein 7 [4]. Chan Li et al. reported a kidney recovery rate of about 65% in a cohort of 1,835 patients, which is lower than the 80% recovery seen in other causes, typically occurring within a median of 10 days [5]. Over time, there has been a decrease in the incidence and severity of AKI requiring dialysis during the COVID-19 pandemic, with the first wave showing the most severe cases [6]. This trend indicated a reduction in the virus’s virulence due to mutations and the impact of vaccination in boosting collective immunity [2]. Improved management strategies have also likely contributed to this positive outcome [2].

AKI in COVID-19 is associated with several laboratory anomalies, notably proteinuria, which emerged as an independent predictor of disease progression [7]. A recent study on predominantly omicron variant developed a risk assessment model identifying seven key parameters across comorbidities, biochemical indicators, inflammatory markers, and vaccine dosage. Patients in the proteinuria group were generally older, had a higher proportion of men, and elevated body mass index. This group also experienced more complications, higher levels of uric acid and C-reactive protein, and a greater incidence of unvaccinated individuals, along with fewer patients receiving three vaccination doses [8]. Alterations in the hematological parameters can also be indicative of patient outcomes, with the red cell distribution width to monocyte percentage ratio serving as a valuable predictive marker for CKD patients with COVID-19 [9].

Kidney biopsies were performed only if they will affect immediate management and deferred for patients on empiric treatment. Kidney biopsies in COVID-19 patients reveal various disorders, primarily *de novo* collapsing glomerulopathy (COVAN) and acute tubular injury, with less common findings including thrombotic microangiopathy, focal segmental glomerulosclerosis, minimal change disease, membranous nephropathy, IgA nephropathy, crescentic glomerulonephritis, lupus nephritis, and anti-GBM disease [10,11]. COVAN, first linked to COVID-19, is notably common among individuals of African descent [12]. Unlike HIVAN, no viral particles have been found in affected kidney cells, indicating that COVAN may result from a systemic response to the infection rather than direct viral effects [13]. Evidence suggests that COVAN may be driven by a cytokine storm, particularly among individuals with certain genetic predispositions, such as those carrying variants of the APOL1 gene [13]. Recent observations suggest that while kidney disease post COVID-19 vaccination is rare, there have been cases of AKI following vaccination, with crescentic glomerulonephritis being the main pathological finding. Other diagnoses included acute tubular injury, IgA nephropathy, ANCA-associated vasculitis, minimal change disease, and thrombotic microangiopathy [4].

Supportive therapy for COVID-19 related AKI focuses on maintaining fluid balance with isotonic or balanced crystalloids and avoiding nephrotoxic medications [14]. While various drugs have been explored, none demonstrate direct renoprotection. Remdesivir may aid recovery in moderate to severe cases whereas nirmatrelvir-ritonavir and molnupiravir reduced hospitalization and mortality in CKD patients, primarily through viral suppression [15,16]. Hydroxychloroquine and azithromycin lack proven benefits and may increase renal risks, especially in severe cases [4,17]. Immunomodulatory therapies like corticosteroids and interleukin-6 receptor antagonists (Tocilizumab) improved mortality rates by reducing systemic inflammation, which may indirectly protect the kidneys [18]. Similarly, Baricitinib, another IL-6 receptor inhibitor, shows antiviral efficacy by targeting the JAK-STAT signaling pathway [19]. Experimental studies indicate that JAK/STAT/APOL1 signaling reduces podocyte viability in kidney organoids, but baricitinib treatment can mitigate this effect [20]. New oral antiviral medications such as simnotrelvir/ritonavir and VV116 have emerged, with the former showing favorable effectiveness in clinical trials [21,22]. Finally, while modulating the renin-angiotensin system has been considered, randomized trials show no benefit from ACE inhibitors or angiotensin receptor antagonist (ARB) in hospitalized patients [23]. Some studies suggest that these medications may increase ACE2 expression, raising concerns about the potential for the virus to exploit this for entry into cells [24]. Leading cardiology organizations have recommended continuing ACEI/ARB therapy in patients with preexisting conditions like hypertension and heart disease [25].

For patients requiring renal replacement therapy, the relationship between cytokine storm and COVID-19 complications suggest a potential benefit of convective modes of continuous renal replacement therapy (CRRT) for more effective removal of inflammatory cytokines, although evidence supporting this approach remains inconclusive [26,27]. Patients with COVID-19 were increasingly noted to be hypercoagulable and as a preventative measure, routine anticoagulation of the CRRT circuit with regional citrate anticoagulation was the preferred method due to its efficacy and reduced bleeding risk [28]. In tandem with these renal therapies, blood purification methods such as coupled plasma filtration adsorption, Cytosorb hemadsorption, high cutoff membranes, and Oxiris membranes have been investigated, although clinical trials have not consistently demonstrated survival benefits from these interventions [29]. Extracorporeal membrane oxygenation (ECMO) was used for acute respiratory failure, particularly in COVID-19 related acute respiratory distress syndrome [29]. Management of ECMO was complicated by the need for CRRT in most cases, which increases mortality risk for intensive care unit patients with AKI [30]. Acute peritoneal dialysis (PD) has been utilized effectively, employing either manual exchanges or cyclers. Line extensions were utilized to minimize unnecessary exposure to staff. However, challenges such as staff unfamiliarity with PD, patient positioning needs, and complications related to fluid balance, especially in obese patients, had to be addressed which limited their use [31].

There is progression of kidney injury post-COVID and unresolved tubular lesions, vascular damage, and podocytopathies are mechanisms behind progression of CKD [2]. A low-grade inflammatory response might contribute to kidney decline, linking acute to chronic inflammation post-SARS-CoV-2 infection *via* damage-associated molecular patterns [2]. This progression can also be associated with long coronavirus disease, commonly known as post-COVID-19 or post-acute COVID-19 syndrome, which describes symptoms and effects that persist for at least 3 months following a COVID-19 infection and cannot be attributed to other diagnoses with a higher risk observed among hospitalized patients and those needing intensive care [32]. The potential mechanisms of long COVID include immune-mediated injury involving podocytes and tubular epithelial cells, diffuse endothelial injury, and increased tubulo-interstitial fibrosis driven by profibrotic signaling pathways [33]. Identifying patients at risk for CKD progression is essential for implementing renal-protective strategies to reduce cardiorenal risk and improve outcomes for this vulnerable population. But at the same time, vaccination is protective against renal injury and progression. Research has shown that higher doses of the COVID-19 vaccine not only reduce infection and hospitalization but also reduce the risk of long COVID as well as proteinuria incidence, emphasizing the ongoing importance of COVID-19 vaccinations, especially booster doses [34].

For kidney transplant recipients, adjustments to immunosuppressive therapies were essential [35]. Common practices involve decreasing or stopping antimetabolites entirely, and for severe infections, the cessation of calcineurin inhibitors was necessary [36]. Observational studies suggest that these adjustments did not significantly raise organ rejection risks [36,37]. Surprisingly, not all transplant patients with COVID-19 needed hospitalization [38]. Many were closely monitored as outpatients, allowing them to recover at home while regularly checking their symptoms and clinical status [39]. Notably, there have been no reported cases of donor-derived COVID-19 transmission in the United States [40]. For nephrologists treating glomerular diseases, the challenge is balancing immunosuppressive therapy with potential kidney risks. Guidelines suggest starting immunosuppression in high-risk patients while considering deferring it for those at lower risk until COVID-19 rates decline. If intravenous immunosuppression started, transitioning to home infusions or oral medications was advised, and telemedicine follow-up care was utilized. Kidney biopsies for suspected glomerular disease were performed only if they will affect immediate management and deferred for patients on empiric treatment [41].

The COVID-19 has significantly impacted healthcare workers, especially nephrologists and nurses, increasing their risks and emphasizing their crucial roles. There was a surge in demand for renal replacement therapy amid limited resources and trained personnel. During the initial surge of COVID-19 in late 2021, there were significant concerns regarding the potential shortage of CRRT machines. In response, various protocols were established at both institutional and national levels to address scarcity and facilitate the efficient redistribution of resources. Modifications were also made to clinical outcome predictors. Notably, a 4-organ Sequential Organ Failure Assessment (SOFA) score was studied as a pragmatic alternative to the full SOFA scoring system for triaging CRRT allocation which aimed to optimize resource use during a period of heightened demand [42].

Nephrologists showed remarkable resilience in managing critically ill patients despite supply shortages, leading to better outcomes for many vulnerable individuals. The COVID-19 pandemic has highlighted the vulnerabilities of dialysis patients, who are at a heightened risk for severe complications and mortality due to multiple comorbidities and potential immunocompromised states. In response to the pandemic, significant regulatory changes have been implemented allowing for expanded use of telemedicine and deferring in-person visits [43]. To minimize exposure, dialysis centers have implemented strict measures, including quarantine protocols, and the use of personal protective equipment. Infected patients were isolated either by shift or by center, with adjustments made to waiting areas to ensure social distancing. Consequently, advancing home dialysis options was encouraged [44,45]. The COVID-19 public health emergency significantly advanced telehealth, enabling remote patient evaluations and reducing travel and exposure risks. It has been especially beneficial for outpatient dialysis and kidney transplantation, with many programs shifting to virtual visits. Yet, issues like education gaps, infrastructure inadequacies, and lack of support hinder broader adoption of telehealth and home therapies.

## Data Availability

Data sharing is not applicable to this article as no new data were created or analyzed in this study.
